# Effectiveness of Schroth exercises during bracing in adolescent idiopathic scoliosis: results from a preliminary study—SOSORT Award 2017 Winner

**DOI:** 10.1186/s13013-017-0139-6

**Published:** 2017-10-16

**Authors:** Kenny Yat Hong Kwan, Aldous C.S. Cheng, Hui Yu Koh, Alice Y.Y. Chiu, Kenneth Man Chee Cheung

**Affiliations:** 1 Department of Orthopaedics and Traumatology, Li Ka Shing Faculty of Medicine, The University of Hong Kong, Pokfulam, Hong Kong; 2 Department of Physiotherapy, Duchess of Kent Children’s Hospital, Sandy Bay, Hong Kong

**Keywords:** Schroth, Scoliosis-specific exercise, Adolescent idiopathic scoliosis, Bracing, Curve progression, Conservative management

## Abstract

**Background:**

Bracing has been shown to decrease significantly the progression of high-risk curves to the threshold for surgery in patients with adolescent idiopathic scoliosis (AIS), but the treatment failure rate remains high. There is evidence to suggest that Schroth scoliosis-specific exercises can slow progression in mild scoliosis. The aim of this study was to evaluate the efficacy of Schroth exercises in AIS patients with high-risk curves during bracing.

**Methods:**

A prospective, historical cohort-matched study was carried out. Patients diagnosed with AIS who fulfilled the Scoliosis Research Society (SRS) criteria for bracing were recruited to receive Schroth exercises during bracing. An outpatient-based Schroth program was given. Data for these patients were compared with a 1:1 matched historical control group who were treated with bracing alone. The assessor and statistician were blinded. Radiographic progression, truncal shift, and SRS-22r scores were compared between cases and controls.

**Results:**

Twenty-four patients (5 males and 19 females, mean age 12.3 ± 1.4 years) were included in the exercise group, and 24 patients (mean age 11.8 ± 1.1 years) were matched in the control group. The mean follow-up period for the exercise group was 18.1 ± 6.2 months. In the exercise group, spinal deformity improved in 17% of patients (Cobb angle improvement of ≥ 6°), worsened in 21% (Cobb angle increases of ≥ 6°), and remained stable in 62%. In the control group, 4% improved, 50% worsened, and 46% remained stable. In the subgroup analysis, 31% of patients who were compliant (13 cases) improved, 69% remained static, and none had worsened, while in the non-compliant group (11 cases), none had improved, 46% worsened, and 46% remained stable. Analysis of the secondary outcomes showed improvement of the truncal shift, angle of trunk rotation, the SRS function domain, and total scores in favor of the exercise group.

**Conclusion:**

This is the first study to investigate the effects of Schroth exercises on AIS patients during bracing. Our findings from this preliminary study showed that Schroth exercise during bracing was superior to bracing alone in improving Cobb angles, trunk rotation, and QOL scores. Furthermore, those who were compliant with the exercise program had a higher rate of Cobb angle improvement. The results of this study form the basis for a randomized controlled trial to evaluate the effect of Schroth exercises during bracing in AIS.

**Trial registration:**

HKUCTR-2226. Registered 22 June 2017 (retrospectively registered)

## Background

The aim of treatment of adolescent idiopathic scoliosis (AIS) is to prevent curve progression to 50°, beyond which there is a risk of continued progression in adulthood. Surgery is therefore usually recommended if the curve reaches 50° during adolescence. Treatment with rigid bracing has recently been shown in the Bracing in Adolescent Idiopathic Scoliosis Trial (BRAIST) to decrease significantly the progression of high-risk curves to the threshold for surgery [1] and is the most widely accepted form of treatment for the prevention of curve progression worldwide. Nonetheless, the rate of treatment success was reported to be 72%, suggesting a proportion of patients will still need to undergo surgery despite bracing.

The standard of care for non-operative management of scoliosis varies widely between North America and Europe [2, 3], and the use of physiotherapy scoliosis-specific exercises (PSSE) is not universally established or accepted. Exercise therapy is well-received by patients and parents [4], and several systematic reviews and randomized controlled trials have reported the positive effects of PSSE on slowing curve progression, improving cosmetic appearance, and quality of life (QOL) outcomes [5–7]. Nonetheless, these studies consisted of a heterogeneous population receiving mixed treatment regimens, various stages of skeletal maturity, and non-standardized outcome measures. Thus, the effect of PSSE on curve progression in the clinical scenario where the curves are at the highest risk of progression has remained unclear.

The Schroth method is the most widely studied and used PSSE approach. It consists of three-dimensional principles of correction, namely auto-elongation, deflection, derotation, rotational breathing, and stabilization [8]. It uses specific rotational angular breathing for vertebral and rib cage derotation, with muscle activation and mobilization. It emphasizes postural corrections throughout the day to change habitual postures and improve alignment, pain, and progression. The Schroth method exercises are curve pattern specific and can be applied in ordinary daily activity, thereby allowing the patients to spend more time in leisure activities and to live a normal life [9].

The Society on Scoliosis Orthopaedic and Rehabilitation Treatment (SOSORT) guidelines recommend the use of PSSE as a stand-alone therapy, add-on to bracing, and during the postoperative period [2]. Romano et al. [10] found that exercises produced a significant increase in the mechanical forces exerted at rest by the fiberglass brace in AIS patients. The positive effects of PSSE can exert its maximal clinical benefit if it improves the outcome of bracing in patients with the highest risk for progression. An improvement of the treatment success of bracing will decrease the rate of surgical interventions in AIS patients.

Therefore, the aim of this study was to assess prospectively the effect of Schroth exercise on curve progression, appearance, and QOL in AIS patients with high-risk curves during bracing.

## Methods

### Study design

A prospective, historical cohort-matched study was conducted. The study was done in compliance with the principles of Good Clinical Practice and the Declaration of Helsinki. The local Institutional Review Board approved the study protocol (Reference Number: UW 17-136). All patients’ parents or legal guardians gave written informed consent.

### Patient enrolment

Consecutive patients with AIS who met the Scoliosis Research Society (SRS) criteria for bracing [11] and received bracing were enrolled for the study. Inclusion criteria were as follows: age of 10 to 15 years, skeletal immaturity (defined on the Risser scale [12] as 0–2 inclusively or R6 U5 score or below on the Distal Radius Ulna Classification [13]), a Cobb angle for the largest curve of 25° to 40° [14], and ability to attend all the physiotherapy sessions. Exclusion criteria were diagnoses other than AIS, disabilities or systemic illnesses preventing exercise performance, and any other previous treatment for AIS.

### Study interventions

**Fig. 1:**
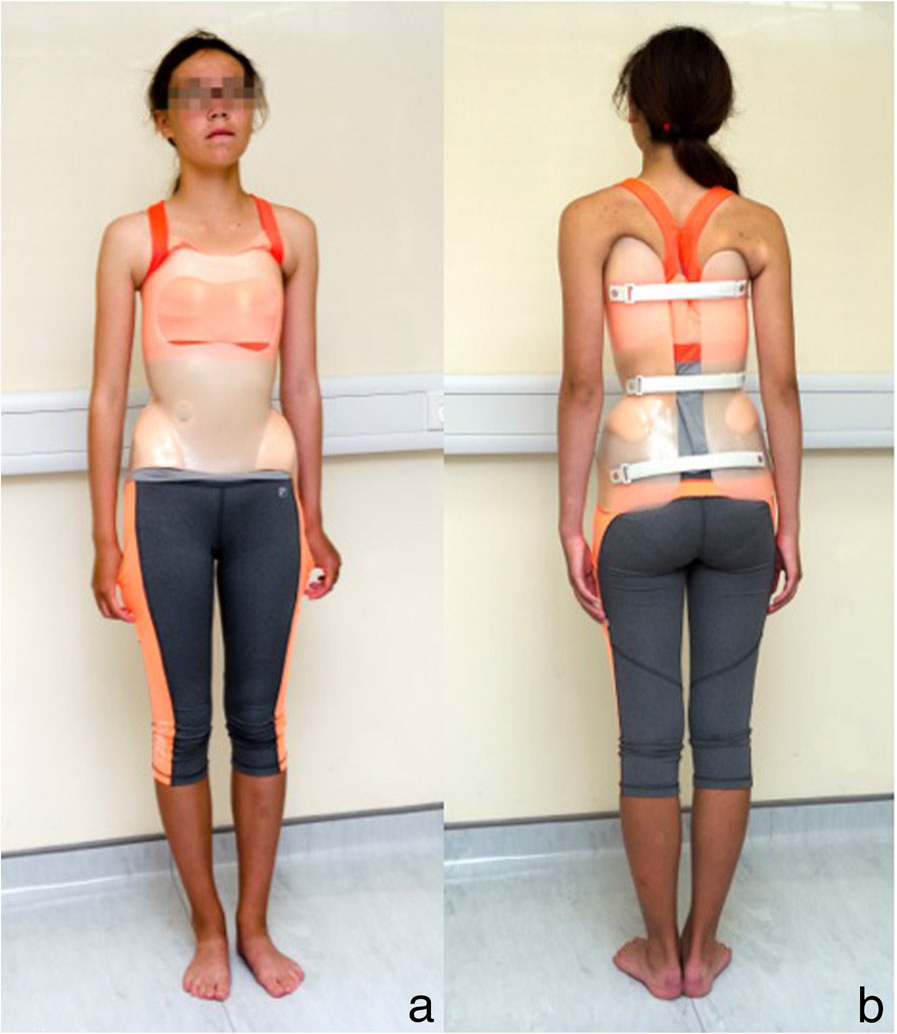
A typical underarm orthosis for curve whose apex is at T7 or below, illustrating the view from the anterior (**a**) and posterior (**b**)

All patients received a rigid underarm orthosis (Fig. 1), prescribed to be worn for a minimum of 18 h per day. The SOSORT Management for bracing guidelines for the physicians, orthotists, and physiotherapists were followed [15].

Schroth-certified therapist was involved and provided all the therapy sessions. No other treatments were advised during the study period.

### Experimental group

**Fig. 2:**
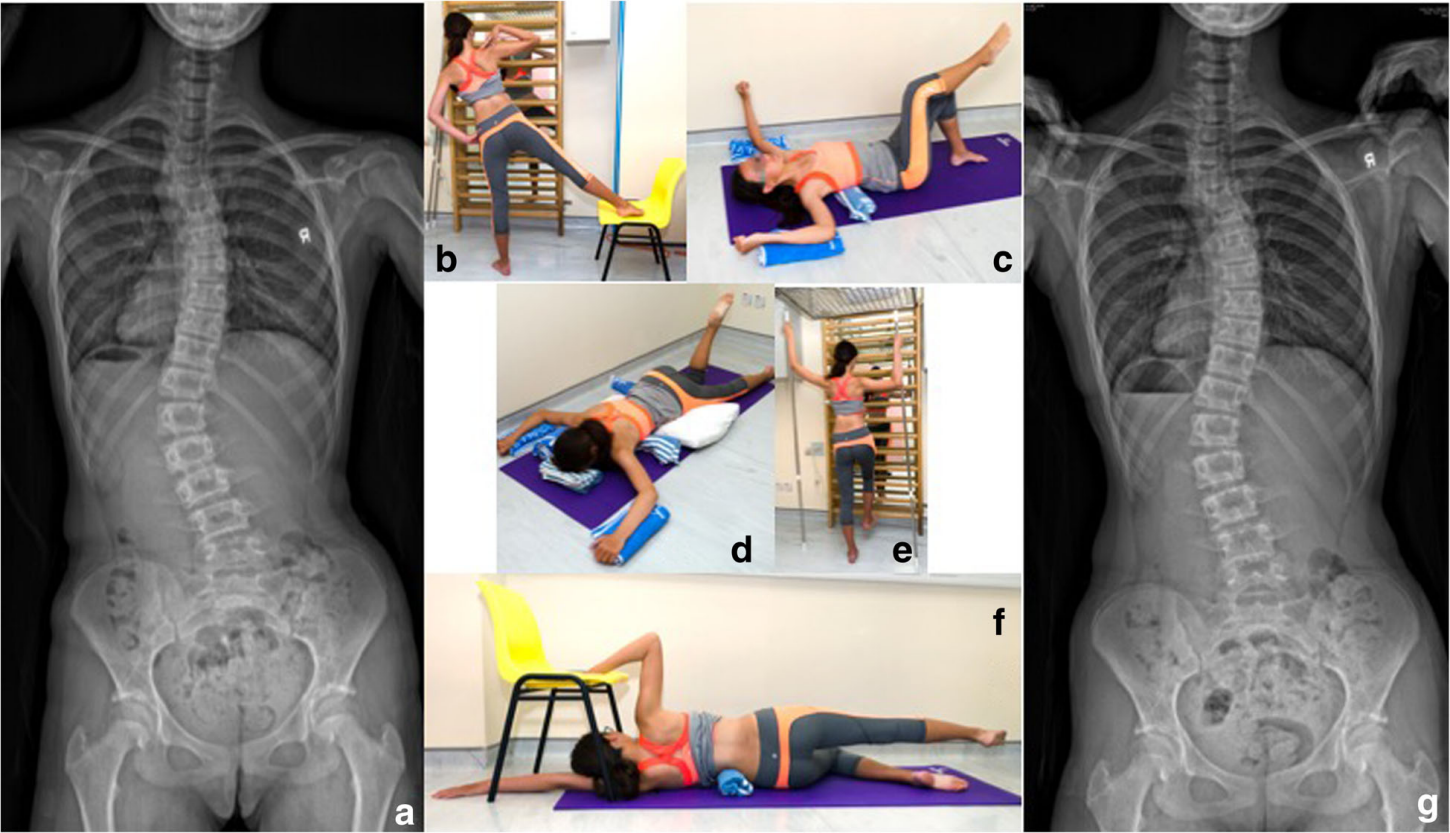
An illustrative case demonstrating five sets of exercises prescribed to patients. **a** Radiograph pre-treatment showing a thoracolumbar major curve from T11 to L3 with a Cobb angle of 40°. Exercises shown here include the following: **b** muscle cylinder in standing, **c** shoulder counter-traction in supine, **d** shoulder counter-traction in prone, **e** shoulder counter-traction in standing with two poles, and (**f**) shoulder counter-traction in side-lying. **g** Radiograph at the completion of training showing an improvement of Cobb angle to 34.6°

The Schroth exercise intervention consisted of an individualized 8-week outpatient program that included four initial private training sessions, once every 2 weeks, where exercises were taught to the patient and their caregivers. A home exercise program was instituted thereafter, and patients were required to return for supervised sessions once every 2 months. Exercises were given in a pamphlet with a description of the corrective movements required, the curve type for which they were recommended, and digital photos of all the exercises taken during their private sessions which they were expected to perform at home. Figure 2 illustrates a case example of a specific curve type and the exercises that were prescribed.

Compliance was monitored and verified daily by their caregivers and during the review sessions by the therapists. During these sessions, adequate exercise performance was assessed using a checklist. Attendance was calculated as a percentage of the prescribed visits attended and compliance as a percentage of the prescribed exercises completed to the therapists’ satisfaction. Compliance was defined as > 80% of attendance of therapy sessions and completion of the prescribed home exercise program at least five out of 7 days per week.

### Control group

A 1:1 historical cohort who was treated in the same institute with bracing only and matched for age, gender, skeletal maturity, and curve magnitude was used as a control group.

### Outcome measures

The outcome measures were radiological deformities (primary outcome), clinical deformities, and QOL scores (secondary outcomes).

Cobb angles of all the major structural curves were measured on a standing posterior-anterior full-spine radiograph. Radiographic definitions of change were based on the SOSORT and SRS non-operative committee consensus [16]: improvement as 6° or more, unchanged as ± 5°, and progressed as 6° or more.

Clinical deformity was recorded in terms of truncal shift and angle of trunk rotation (ATR). The Bunnell scoliometer was used to measure the ATR, i.e., the angle between the horizontal plane and a plane across the posterior aspect of the trunk, of the hump in the main structural curve with the patient bending forward [17].

The SRS-22 questionnaire is a scoliosis-related QOL questionnaire that assesses five domains: function, pain, self-image, mental health (five questions each), and satisfaction with care (two questions). Each question is scored from 1 to 5, where 1 is the worst and 5 the best. The Chinese version was administered, which had been validated [18].

### Adverse effects

Patients were asked to record any serious symptoms or events they experienced during the study.

### Statistics

Student’s paired *t* test (*p* < 0.05) was made for each of the outcome measures. Sub-analysis was performed within the experimental group to study the effects of compliance. The data were analyzed using SPSS 21.0 software.

## Results

### Subjects

Twenty-four (5 males and 19 females) were recruited into the experimental group, and 24 patients were matched in the control group. Both groups did not differ at baseline for age, gender, Risser sign, and magnitude of the main structural curves (Table 1). The mean age was 12.3 ± 1.4 years in the experimental group and 11.8 ± 1.1 years in the control group. The experimental and control groups had a mean follow-up period of 18.1 ± 6.2 and 38.8 ± 11 months, respectively.

**Table 1:** Baseline characteristics of the scoliosis-specific exercise and the historical-matched cohort groups

	SSE group	cohort group
Number of subjects	24	24
Age (mean/SD)	12.3 (10–14)/1.4	11.83 (10–14)/1.1
Gender (%)		
Female	79.2	79.2
Male	20.8	20.8
Risser sign at the start of treatment (%)		
0–1	54.2	79.2
2	29.2	16.7
≥ 3	16.6	4.2
Region of largest curve (%)		
Thoracic	20.8	33.3
Thoracolumbar/lumbar	79.2	66.7
Period of re-assessment/months (mean/SD)	18.1/6.2	38.75/11

*SSE* scoliosis specific exercise, *SD* standard deviation

### Effects of the interventions

After training, the spinal deformity improved in 17% of the patients in the experimental group (Cobb angle decreases by 6° or more), worsened in 21% (Cobb angle increases by 6° or more), and remained stable in 62% (Cobb angle was ± 5°). In the control group, 4% improved, 50% worsened, and 46% remained stable.

After training, the mean ATR improved from 9.43° ± 3.27° to 8.45° ± 3.45°, although it did not reach statistical significance (*p* = 0.08), and it remained stable in the control group. There was no statistical significant difference in the mean truncal shift in the experimental and the control groups.

For the SRS-22 domains, high scores were noted at the baseline for both groups (mean of 4.25 ± 0.38 and 4.10 ± 0.52 out of 5). Statistical significant improvements were found in the experimental group in the function domain (4.60 ± 0.44 to 4.76 ± 0.33, *p* = 0.05) and the total score (4.25 ± 0.38 to 4.45 ± 0.34, *p* = 0.04) whereas changes in the other domains did not reach statistical significance. No significant changes were noted for the control group in any of the domains or the total score.

### Effects of compliance

Brace compliance was rated as good in 70.8% in the experimental group and 79.2% in the historical cohort group. In the experimental group, 76.9% of patients who were compliant to the Schroth exercises had good bracing compliance, whereas only 63.6% of those who were non-compliant to the exercises had good bracing compliance.

In the experimental group, 13 patients were found to be compliant to Schroth exercises according to our definition above, and 11 patients did not meet this criterion. Compliance was strongly associated with curve improvement (31 vs 0%) and negatively associated with curve progression (0 vs 46%). Compliance was also positively associated with improvements in truncal shift from 11.87 ± 8.16 to 7.09 ± 6.41 mm (*p* = 0.01) and ATR from 10.15° ± 3.65° to 8.69° ± 3.01° (*p* = 0.043).

### Adverse effects

No adverse effects were noted during the study period.

## Discussion

This is the first prospective study investigating the effect of Schroth exercises on curve progression, topographical changes, and SRS-22 scores in AIS patients during bracing. The findings of this study show that Schroth exercises during bracing can increase the proportion of patients with Cobb angle improvement ≥ 6° by 6% compared with bracing alone. In addition, our results suggest that 20% more patients have improved Cobb angles of ≥ 6° if they are compliant with Schroth exercises during bracing compared with bracing alone. However, the outcomes of non-compliant patients were slight worse than the historical cohort, which might partly due to a worse compliance to brace treatment in this group.

Although previous studies have demonstrated the superiority of scoliosis-specific exercises in reducing curve progression, they were performed in a population undergoing conservative treatment for mild AIS only [19–23]. Furthermore, their data cannot be generalized to rehabilitation under other clinical scenarios, such as during bracing or after surgical correction. This preliminary study focused on a group of high-risk patients who were all treated with bracing. The usual intervention after treatment failure in these patients would be surgical correction and fusion and was recently reported in the BRAIST to be 25–28% [1]. Thus, any further treatment during bracing that can improve the outcome can lower the surgical rate. We show that the efficacy of bracing can be further improved by the addition of Schroth exercises with a strong compliance-response relationship.

Although there was a trend towards ATR reduction in the experimental group, it did not reach statistical significance in our study. All previous studies that reported ATR showed a decrease after scoliosis-specific exercises ranging from 0.33° to 4.23° [24, 25]. Schroth exercises have been shown to improve the cosmetic appearance in children, demonstrated in some studies to decrease the height of the hump [26], and improving waist asymmetry [27]. Although we cannot make a definite conclusion from our results, a more reliable and valid measure of objective cosmetic changes needs to be included in future studies.

The effect of the treatment on the SRS-22 scores shows that Schroth exercises improve the overall QOL in AIS patients during bracing. However, it is now increasingly noted that the SRS-22 questionnaire was designed to study the effects of surgery in AIS and suffers a ceiling effect in conservative treatments [7, 28, 29]. The high scores reported at the baseline therefore limit the ability of this questionnaire to measure large improvements. Different tools, such as SRS-7, Trunk Appearance Perception Scale (TAPS), Patient-Reported Outcomes Measurement Information System (PROMIS), and computer adaptive testing (CAT) instruments, may be administered together in future studies to detect clinically significant differences in their function and QOL. Currently, no alternative validated evaluating tools are available.

Our findings suggest that administering Schroth exercise program as an outpatient is feasible and has a reasonable compliance. These results are consistent with earlier findings that a physiotherapist-supervised Schroth exercise program is superior to a home-based program or no treatment [25]. In their study, the supervised program consisted of 18 sessions (1.5 h a day, 3 days a week) for 6 weeks. However, this would be too demanding for patients in this locality, and we predicted this would have deleterious effects on the study enrolment, the attendance, and compliance rate. We therefore modified the protocol to four sessions (1 h per session fortnightly) for 8 weeks. This was a compromise between maintaining adequate supervision and minimalizing disruption to the patients’ and their families’ lives.

The study has several limitations. First, it was a historical cohort comparison but every effort has been made to ensure the two groups are compatible by age, gender, and curve magnitude matching. However, there was a difference in the follow-up period between the two groups. At the time of analysis, all patients in the experimental group had a minimum of 12 months of follow-up, but some patients in the historical cohort had already completed treatment. Nonetheless, we felt this cohort provided a reasonable control since the only difference in intervention between the groups was the addition of Schroth training. Secondly, exercise compliance and adherence to treatment could not be fully assured, although the patients’ diaries were checked, and full engagement of the caregivers ensured accurate data collection. Thirdly, although brace compliance between the two groups was comparable, sub-analysis based on exercise compliance found a difference in brace compliance between the groups and historical control. Hence, these results should be interpreted with caution. Fourthly, the therapists could not be blinded to the treatment group, although the analyses were done by an independent assessor. Finally, the sample size in the sub-analysis for compliance is small.

## Conclusions

This is the first study to investigate the effects of Schroth exercises during bracing in patients with a high risk of curve progression. The findings from this preliminary study suggest that Schroth exercises during bracing can further improve the Cobb angle compared with bracing alone and compliance is associated with greater benefit. Based on the results of this study and using the current protocol, appropriate sample size calculation and attrition rate can be performed for a large-scale trial. Given the promising findings, a prospective, randomized-controlled trial to evaluate the effect of Schroth exercises during brace treatment in AIS patients is now warranted.

## Abbreviations

AIS: Adolescent idiopathic scoliosis
ATR: Angle trunk rotation
BRAIST: Bracing in Adolescent Idiopathic Scoliosis Trial
CAT: Computer adaptive testing
PROMIS: Patient-Reported Outcomes Measurement Information System
PSSE: Physiotherapy scoliosis-specific exercises
QOL: Quality of life
SOSORT: Society on Scoliosis Orthopaedic and Rehabilitation Treatment
SRS: Scoliosis Research Society
TAPS: Trunk Appearance Perception Scale
